# Ethanol extract from *Astilbe chinensis* inflorescence suppresses inflammation in macrophages and growth of oral pathogenic bacteria

**DOI:** 10.1371/journal.pone.0306543

**Published:** 2024-07-03

**Authors:** Jong Min Han, Ina Yun, Kyung Mi Yang, Hye-Sung Kim, Young-Youn Kim, Wonsik Jeong, Seong Su Hong, Inseong Hwang

**Affiliations:** 1 DOCSmedi OralBiome Co. Ltd., Goyang-si, Republic of Korea; 2 Apple Tree Institute of Biomedical Science, Apple Tree Medical Foundation, Goyang-si, Gyeonggi-do, Republic of Korea; 3 Bio Industry Department, Gyeonggido Business & Science Accelerator (GBSA), Suwon-si, Gyeonggi-do, Republic of Korea; Gachon University College of Korean Medicine, REPUBLIC OF KOREA

## Abstract

Chronic oral inflammation and biofilm-mediated infections drive diseases such as dental caries and periodontitis. This study investigated the anti-inflammatory and antibacterial potential of an ethanol extract from *Astilbe chinensis* inflorescence (GA-13-6) as a prominent candidate for natural complex substances (NCS) with therapeutic potential. In LPS-stimulated RAW 264.7 macrophages, GA-13-6 significantly suppressed proinflammatory mediators, including interleukin-6 (IL-6), tumor necrosis factor (TNF), and nitric oxide (NO), surpassing purified astilbin, a known bioactive compound found in *A*. *chinensis*. Furthermore, GA-13-6 downregulated the expression of cyclooxygenase-2 (COX2) and inducible nitric oxide synthase (iNOS), indicating an inhibitory effect on the inflammatory cascade. Remarkably, GA-13-6 exhibited selective antibacterial activity against *Streptococcus mutans*, *Streptococcus sanguinis*, and *Porphyromonas gingivalis*, key players in dental caries and periodontitis, respectively. These findings suggest that complex GA-13-6 holds the potential for the treatment or prevention of periodontal and dental diseases, as well as various other inflammation-related conditions, while averting the induction of antibiotic resistance.

## Introduction

Periodontal diseases and dental caries rank as the two primary causes of tooth loss [1]. Periodontal diseases, such as gingivitis and periodontitis, arise from the accumulation of polymicrobial infections affecting structures supporting the teeth, such as gingiva (the gums), periodontal ligament, and alveolar bone. Gingivitis, the initial stage of chronic periodontitis, involves localized reversible inflammation of the gingiva triggered by bacteria within a microbial biofilm [2]. One of the significant pathogens within the biofilm includes *P*. *gingivalis*, an anaerobic, asaccharolytic, immotile Gram-negative bacterium typically found in subgingival pockets [3,4]. Dental caries, commonly referred to as cavities, result from biofilm-dependent tooth decay due to the breakdown of tooth hard tissues (enamel, dentin, and cementum) caused by acidic by-products (e.g., lactic acid) fermented by saccharolytic bacteria, such as *S*. *mutans* and *S*. *sanguinis* [5]. *S*. *mutans* and *S*. *sanguinis*, both Gram-positive bacteria belonging to the phylum Firmicutes, adhere to the supragingival delamination layer of teeth. *S*. *mutans*, with higher acid tolerance than *S*. *sanguinis*, is considered a major etiologic agent in disease onset [6]. Although *S*. *sanguinis* typically functions as a commensal bacterium in a healthy oral cavity, dysbiosis in the oral microbiota can transform it into an opportunistic pathogen. Hence, there is increasing attention on understanding the ecological balance of oral microflora in the context of widespread diseases. Moreover, these oral pathogens have been linked to systemic diseases [7] including, but not limited to, endocarditis [8] and cardiovascular disease [9,10]. Particularly, *P*. *gingivalis* is recognized for its role in developing cognitive diseases [11–14].

Traditionally, antibiotics have been the primary approach for controlling bacterial growth and infection in the oral cavity, with commonly used examples including amoxicillin, azithromycin, metronidazole, and moxifloxacin [15]. However, the widespread and often unnecessary use of antibiotics is now acknowledged as a significant contributor to disrupting the oral microbiome, leading to dysbiosis and the emergence of antibiotic-resistant bacteria [16]. These resistant strains not only complicate the treatment of oral diseases but also increase the risk of systemic infections and associated morbidity [17]. Given these concerns, there is a growing imperative to develop alternative strategies such as natural products (NP) and natural complex substances (NCS) for managing oral bacterial burden and inflammation that circumvent the emergence of antibiotic resistance [18]. Such strategies have the potential to improve the long-term efficacy of oral disease treatment and safeguard overall health.

*Astilbe chinensis* (Maxim.) Franch. et Savat., a perennial herbaceous plant from the family Saxifragaceae, is found in China, Japan’s Tsushima Island, northeastern Russia, India, and Korea. In South Korea, the plant is abundant across the country. Historically, the underground parts of *A*. *chinensis* have been utilized for both food and medicine, treating ailments such as headaches and bronchitis, and serving as an antipyretic and analgesic remedy [19]. Research indicates that *A*. *chinensis* possesses clinical efficacy in regulating adipogenesis [20] and mitigating metabolic disorders [21]. Notable bioactive compounds in *A*. *chinensis* include astilbic acid [22], astilbin [23], and bergenin [24]. Among these, astilbic acid is renowned for its anti-inflammatory activity in immune cells and animal models [22,25], while astilbin has demonstrated efficacy in improving psoriasis in animal models [26] and preventing the development of osteoarthritis [27].

While previous studies have demonstrated the anti-inflammatory effects of an ethanol extract of *A*. *chinensis* (ACE) [28] and the underground parts of *A*. *chinensis* [25,29], a comparison between its habitats, seasons, and different parts of the plant, as well as the anti-bacterial effect of the ethanol extract on representative oral pathogens, remains unexplored. Here, we for the first time conducted the comparison of the antimicrobial and anti-inflammatory properties of ACEs obtained from three different parts (aerial, underground, inflorescence) across two seasons and four distinct regions. Our findings indicate that GA-13-6, among the ACEs, not only effectively suppresses the activation of inflammatory mediators in LPS-stimulated RAW 264.7 cells but also inhibits the growth of important oral pathogens associated with periodontal diseases and dental caries. Consequently, GA-13-6 emerges as a promising NCS candidate for the prevention and treatment of bacterial-driven oral diseases. Furthermore, its negligible contribution to antibiotic resistance confers a significant advantage over conventional antibiotic therapy, potentially mitigating the development of dysbiosis and associated complications.

## Materials and methods

### Chemicals and reagents

Dulbecco’s modified Eagle’s medium (DMEM) and fetal bovine serum (FBS) were purchased from Corning Inc. (Glendale, AZ). Immobilon^®^-P PVDF membrane, dimethyl sulfoxide (DMSO), ethylenediaminetetraacetic acid (EDTA), tryptic soy broth, yeast extract, hemin, vitamin K1, LPS (*Escherichia coli* O55:B5), and 3-(4,5-dimethylthiazol-2-yl)-2,5-diphenyltetrazolium bromide (MTT) were from Merck (Darmstadt, Germany). Defibrinated sheep blood was purchased from Synergy Innovation (Gyeonggi-do, Korea). Goat anti-mouse IgG (H+L) HRP secondary antibody (Cat. No. 31430), enzyme-linked immunosorbent assay (ELISA) kit, Halt^™^ protease and phosphatase inhibitor cocktail (100×), RIPA buffer, BD Difco^™^ Brain Heart Infusion broth, and Griess reagent were purchased from Thermo Fisher Scientific Korea (Seoul, Korea). The primers used for the qPCR assay were from Bioneer (Daejeon, Korea). The antibodies for β-actin (sc-47778), iNOS (NOS2) (sc-7271), and COX2 (sc-376861) were from Santa Cruz Biotechnology Inc. (Dallas, TX). Astilbin (Cat. No. S3932) was purchased from Selleck Chemicals (Houston, TX). TRIzol^®^ was from Favorgen (Ping-Tung, Taiwan). The BCA assay kit was purchased from Bio-Rad (Hercules, CA).

### Collection of *A*. *chinensis* samples

Three separate parts of *A*. *chinensis* plant (aerial parts, underground parts, and inflorescences) from four separate regions of the Republic of Korea (Gapyeong-gun, Gyunggi-do; Yangsan-si, Gyeongsangnam-do; Suncheon-si, Jeollanam-do; Yanggu-gun, Gangwon-do) were collected in flowering and fruiting seasons in 2018, which were identified by Dr. Jin-Oh Hyun at Northeastern Asia Biodiversity Institute (Table 1). The present study, using wild *A*. *chinensis*, poses no risk of extinction for the species while complying with national and international guidelines and legislation. No special permits were required for sampling, as we restricted collection to public lands excluding protected areas and private property. Voucher specimens were deposited in the Herbarium of the Bio Industry Department, Gyeonggido Business & Science Accelerator (GBSA) in Korea.

**Table 1:** The nucleotide sequences of the primers used in the qPCR study.

Gene	Forward (5’-3’) (bp)	Reverse (5’-3’) (bp)
*Nos2*	ACATCGACCCGTCCACAGTAT (21)	CAGAGGGGTAGGCTTGTCTC(20)
*Cox2*	CTGGTGCCTGGTCTGATGATGTATG (25)	TCTCCTATGAGTATGAGTCTGCTGGTT (27)
*Gapdh*	ACCCAGAAGACTGTGGATGG (20)	CACATTGGGGGTAGGAACAC (20)

### Preparation of an ethanol extract of *A*. *chinensis* (ACE)

We macerated the dried and powdered samples of each plant system with 70% ethanol (1 L per 100 μg of each sample) for 24 h at room temperature to extract them. The resulting extract was used for initial screening or underwent filtration and evaporation *in vacuo* at 40°C, followed by lyophilization. The resulting extract powders were then dissolved in DMSO at a concentration of 100 mg/mL.

### Cell culture and viability assay

We obtained the RAW 264.7 murine macrophage cell line from the American Type Culture Collection (ATCC, MD, USA). The cells were cultured at 37°C in DMEM supplemented with 2 mM glutamine, 100 U/mL penicillin, 100 μg/mL streptomycin, and 10% fetal bovine serum (FBS) under a 5% CO_2_ environment. To assess cell viability, RAW 264.7 cells (3.0×10^5^ cells/well) were seeded in a 6-well plate and incubated for 24 h before experimental interventions. Subsequently, the cells were pre-treated with various concentrations of ACE for 3 h or 16 h before exposure to 3 μL of LPS (100 μg/mL in DMSO) for 3 h. Following treatment, the cells were incubated with MTT (5 mg/mL, 200 μL/well) for 3 h at 37°C. The medium was then removed and 2 mL of DMSO was added to each well to dissolve the formazan crystals in the viable cells. The optical density was measured at a wavelength of 570 nm using Multiskan GO (Thermo Fisher Scientific, CA, USA).

### Measurement of NO and inflammatory cytokines

We seeded RAW 264.7 macrophages at a density of 3.0×10^5^ cells/well in six-well plates and allowed them to incubate for 24 h before initiating experimental interventions. Subsequently, we pre-treated the cells with various concentrations of ACE for either 3 h or 16 h before exposure to LPS for 3 h. Following treatment, we collected the culture media, which was then centrifuged at 13,000 rpm for 3 min at 4°C. We employed the Griess assay to analyze nitrite (NO_2_^-^) levels with an absorbance measured at 548 nm. Specifically, 150 μL of cell culture medium was added to each well of a 96-well plate, followed by the addition of 130 μL of distilled water and 20 μL of Griess reagent (1% sulfanilamide and 0.1% naphthylenediamine dihydrochloride in 2% phosphoric acid). The plate was left to incubate for 10 min at room temperature with agitation. Nitrite production was quantified spectrophotometrically using Multiskan GO (Thermo Fisher Scientific, CA, USA). The levels of TNF and IL-6 were assessed using an ELISA kit by the manufacturer’s instructions.

### Western blotting analysis

Proteins were extracted with RIPA buffer containing Halt^™^ protease and phosphatase inhibitors with 5 mM EDTA. Protein concentrations were determined using a BCA assay kit. Cell lysates containing 20 μg of protein were applied to 10% gels and subsequently transferred to a PVDF membrane. The membrane was then blocked using a 5% skim milk solution, followed by incubation with the primary antibody overnight at 4°C. After washing and re-blocking the membrane, we applied the secondary antibody, diluted 1:5,000 in 5% skim milk, for 1 h. Finally, bands were detected by ChemiDoc^™^ Imaging System (Bio-Rad, CA, USA).

### cDNA synthesis and qPCR

We washed the RNA extracted from the cells once with PBS and then treated it with TRIzol^®^ for prolonged preservation. Reverse transcription of RNA was performed using the Prime Script 1^st^ strand cDNA Synthesis Kit (Takara, Japan), and qPCR was conducted using the Power SYBR Green PCR Master mix (Applied Biosystems, USA) with Exicycler 96 system (Bioneer, Korea). The primers utilized in this study are listed in Table 1.

### Minimum inhibitory concentration (MIC) assay of oral pathogenic bacteria

We obtained strains of *S*. *sanguinis* (KCOM 1070), *S*. *mutans* (KCOM 1054), and *P*. *gingivalis* (KCOM 2796) from the Korean Collection for Oral Microbiology (KCOM) and cultured them under anaerobic conditions (80% N_2_, 10% CO_2_, 10% H_2_). *P*. *gingivalis* was cultivated in KCOM broth supplemented with 5 mg/mL hemin, 1 μg/mL vitamin K1, and 50 mg/mL sterile defibrinated sheep blood at 37°C. Strains of *S*. *sanguinis* and *S*. *mutans* were grown in brain heart infusion (BHI) medium at 37°C. We inoculated a single colony to obtain a freshly saturated culture medium. For the MIC assay, we adjusted the initial optical density at 600 nm (OD_600_) to 0.05 in a 6-well plate using freshly saturated seven-day cultures (*P*. *gingivalis*) and 24-h cultures (*S*. *mutans* and *S*. *sanguinis*). The cells were then treated with GA-13-6 stock (5 mg/mL in ethanol) at final concentrations ranging from 0.008 to 1.0 mg/mL and incubated for five to seven days (*P*. *gingivalis*) and 12 to 24 h (*S*. *mutans and S*. *sanguinis*) before OD_600_ measurement.

### Statistical analysis

Three independent experiments were performed, and the data were analyzed by one-way analysis of variance (ANOVA) with Tukey’s *post-hoc* test for multiple comparisons of more than three different groups.

## Results

### Selection of GA-13-6 based on IL-6 suppression activity

**Fig 1:**
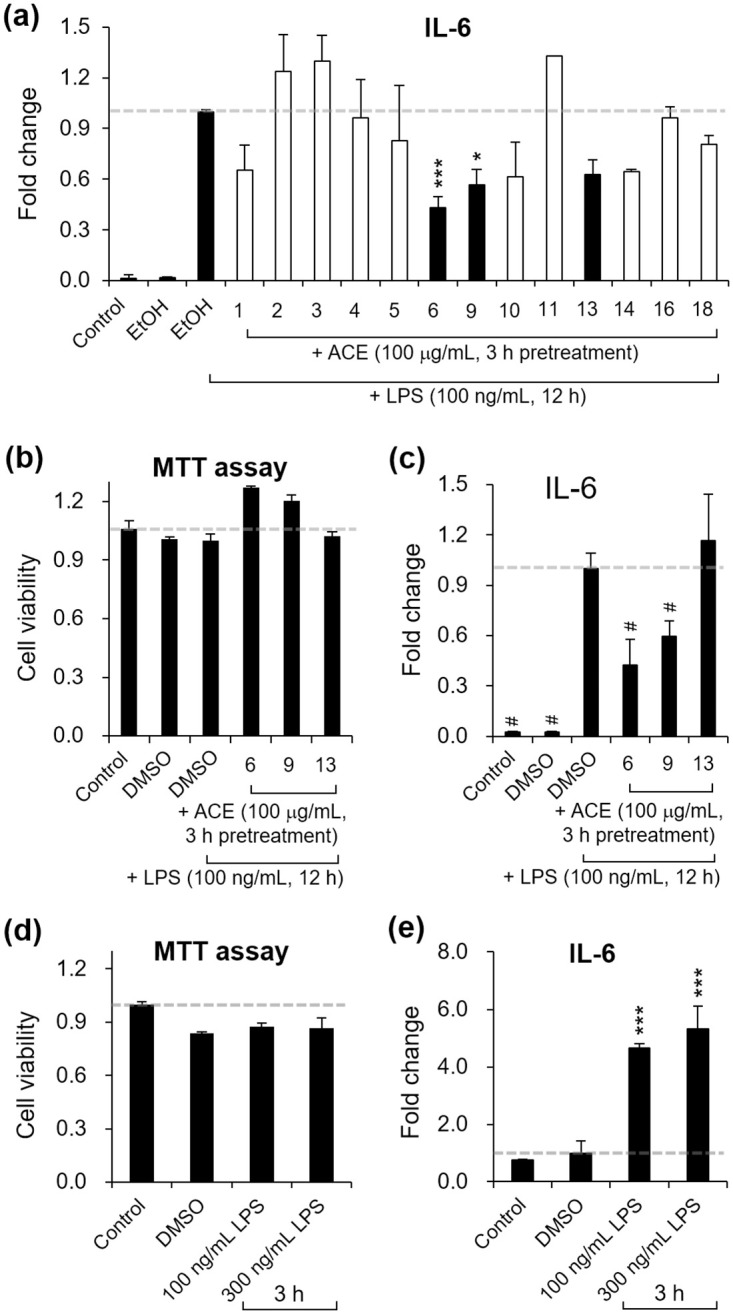
Selection of effective parts of *A*. *chinensis* GA-13-6 in 70% ethanol and DMSO using LPS-induced RAW 264.7 macrophages. (a) RAW 264.7 cells were pre-treated with 100 μg/mL of ACEs from six different samples (GA-13-1, 2, 3, 4, 5, 6, 9, 10, 11, 13, 14, 16, and 18) in ethanol for 3 h, then with 100 ng/mL of LPS in 70% ethanol for 12 h. ACEs that were less effective in suppressing the inflammatory cytokine IL-6 (GA-13-1, 2, 3, 4, 5, 10, 11, 16, and 18) were excluded from further testing. Additionally, GA-13-14 was found to have an insufficient amount for further study. (b) The dried powders of three GA-13-6, 9, and 13, dissolved in DMSO, exhibited no cytotoxicity in cell proliferation assessed by the MTT assay (b) and showed similar effectiveness in IL-6 suppression (c). Treatment with LPS up to 300 ng/mL for 3 h resulted in negligible cytotoxicity (d) while inducing a better fold change in IL-6 levels (e). The data represent the mean ± SD of three or at least two independent experiments. ***p < 0.005 vs ethanol or DMSO control.

We assigned codes GA-13-1 to GA-13-18 to 18 samples (Table 2). Among these, we excluded six samples (GA-13-7, 8, 12, 14, 15, and 17) owing to poor sample quality. Subsequently, we identified ethanolic GA-13-6, GA-13-9, and GA-13-13 as exhibiting relatively high efficacy in suppressing the inflammatory cytokine IL-6 when RAW 264.7 macrophages were pre-treated with 100 μg/mL of ACE for 3 h (Fig 1a). Upon drying and dissolution in DMSO, GA-13-6 maintained the highest efficacy against IL-6 without inducing cytotoxicity (Fig 1b and 1c). We then optimized the duration and the concentration of LPS treatment. Treatment with LPS up to 300 ng/mL for 3 h showed no significant differences in the cell viability (Fig 1d) while yielding enhanced stimulation levels (Fig 1e). Consequently, cells were treated with 300 ng/mL LPS for 3 h for stimulation in subsequent experiments.

**Table 2:** Collection of three different parts of the *Astilbe chinensis* plant (aerial parts, underground parts, and inflorescence) from various regions in flowering and fruiting seasons. GA-13-7, 8, 12, 15, and 17 (gray-colored cells) were excluded based on the initial screening for plant sample quality.

Species	Seasons	Regions (S. Korea)	Parts	Code
***Astilbe chinensis***	Flowering season	Gapyeong-gun, Gyeonggi-do	Aerial	GA-13-1
			Underground	GA-13-2
			Inflorescence	GA-13-3
		Yangsan-si, Gyeongsangnam-do	Aerial	GA-13-4
			Underground	GA-13-5
			Inflorescence	GA-13-6
		Suncheon-si, Jeollanam-do	Aerial	GA-13-7
			Underground	GA-13-8
			Inflorescence	GA-13-9
	Fruiting season	Yanggu-gun, Gangwon-do	Aerial	GA-13-10
			Underground	GA-13-11
			Inflorescence	GA-13-12
		Gapyeong-gun, Gyeonggi-do	Aerial	GA-13-13
			Underground	GA-13-14
			Inflorescence	GA-13-15
		Yangsan-si, Gyeongsangnam-do	Aerial	GA-13-16
			Underground	GA-13-17
			Inflorescence	GA-13-18

### GA-13-6 reduces proinflammatory mediators in LPS-induced RAW 264.7 macrophages

**Fig 2:**
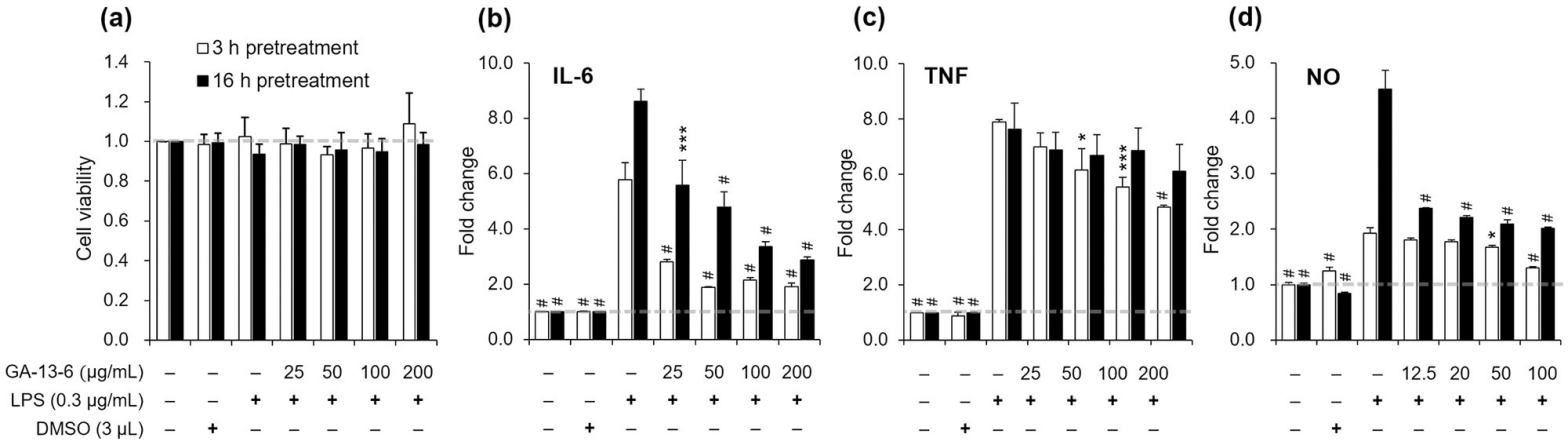
Effect of GA-13-6 on the production of proinflammatory mediators in LPS-induced RAW 264.7 cells. Cells were pre-treatment with GA-13-6 for 3 h (empty bars) or 16 h (filled bars) before treatment with LPS (0.3 μg/mL) for 3 h. Cell viability was assessed by MTT assay (a). Levels of LPS-induced proinflammatory cytokines, IL-6 (b) and TNF (c), were quantified using ELISA. NO levels were measured by Griess assay using culture supernatant (d). *p < 0.05, ***p < 0.005, ^#^*p* < 0.001 vs LPS-only group.

To assess cytotoxicity due to long-term treatment, we employed various concentrations of GA-13-6 to pre-treat cells for 3 h (short-term) and 16 h (long-term) before subjecting them to LPS treatment for 3 h. The results revealed no significant cytotoxicity when cells were pre-treated with GA-13-6 up to 200 μg/mL (Fig 2a). Subsequently, we evaluated the anti-inflammatory activity of GA-13-6 by assessing the suppression of inflammatory markers in macrophages. Upon inducing RAW 264.7 cells with 300 ng/mL of LPS for 3 h, we observed the upregulation of proinflammatory cytokines such as IL-6 (Fig 2b), TNF (Fig 2c), and NO (Fig 2d) in RAW 264.7 cells. However, pre-treatment of cells with GA-13-6 for either 3 h or 16 h, led to dose-dependent suppression of these inductions. Specifically, for IL-6 and TNF, shorter pre-exposure to GA-13-6 resulted in a greater reduction, with IL-6 reduction being more pronounced than TNF (Fig 2b and 2c). Conversely, NO production levels were higher when cells were pre-treated with GA-13-6 for 16 h before LPS stimulation (Fig 2d), resulting in a more substantial dose-dependent reduction in NO. Altogether, pre-treatment with GA-13-6 effectively prevented RAW 264.7 macrophages from activating proinflammatory cytokines upon LPS induction.

### GA-13-6 reduced the mRNA and protein expression levels of COX2 and iNOS

**Fig 3:**
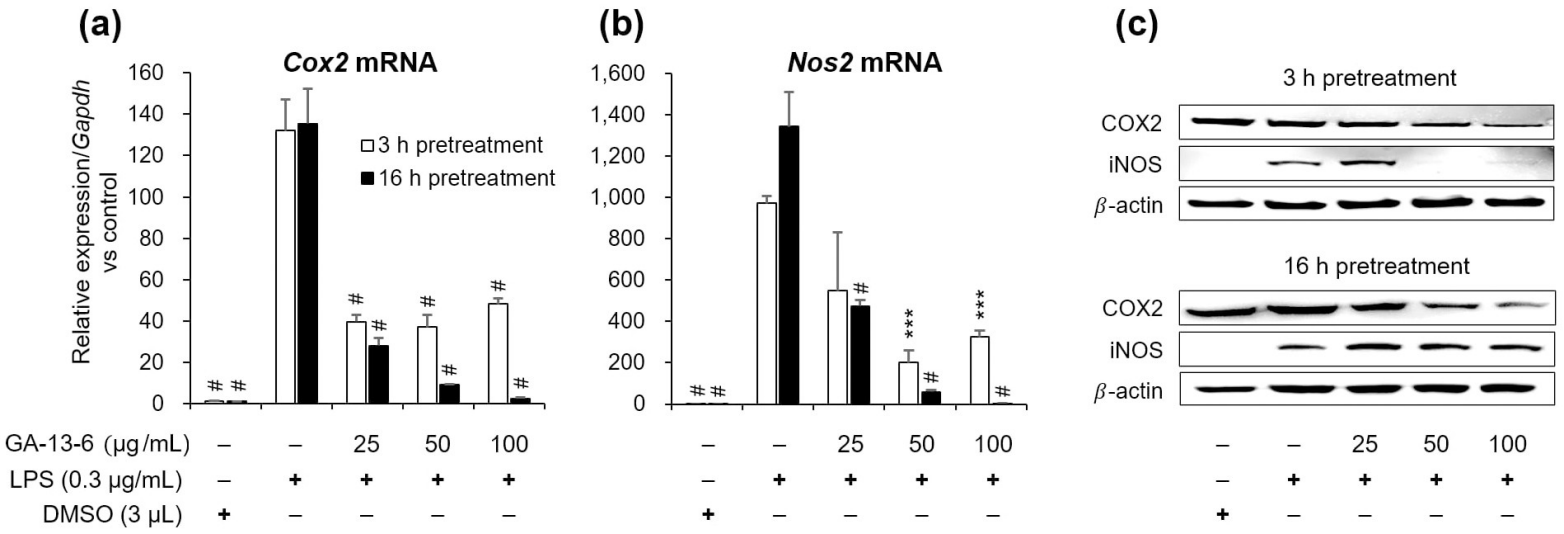
Effect of GA-13-6 on mRNA and protein expression levels of COX2 and iNOS in LPS-induced RAW 264.7 cells. The cells were pre-treated with GA-13-6 (0, 25, 50,100 μg/mL) for 3 h (empty bars) and 16 h (filled bars), respectively, before incubation with LPS (0.3 μg/mL) for 3 h. The mRNA expression levels of *Cox2* (a) and *Nos2* (b) were normalized using *Gapdh* gene. Protein expression levels of COX2 and iNOS (c) were confirmed by Western blotting. ***p < 0.005, ^#^*p* < 0.001 vs LPS-only group.

Both COX2 and iNOS (NOS2) are known as crucial factors associated with the generation of NO in inflammatory reactions [30]. To ascertain whether GA-13-6 inhibits the production of NO and inflammatory cytokines by regulating COX2 and iNOS expression in RAW 264.7 cells, we analyzed the mRNA expression levels of both *Cox2* and *Nos2*. The mRNA expression levels of both genes were significantly suppressed at concentrations above 25 μg/mL of GA-13-6 with either 3 h or 16 h pre-treatment (Fig 3a and 3b). Notably, similar to the NO production pattern depicted in Fig 2d, the mRNA expression levels of both genes induced by LPS stimulation were higher when macrophages were pre-treated with GA-13-6 for 16 h, resulting in a more pronounced reduction of mRNA expression levels in a dose-dependent manner.

We subsequently confirmed that the protein expression levels of COX2 and iNOS decreased when RAW 264.7 macrophages were pre-treated with GA-13-6, as evidenced by Western blotting analysis (Figs 3c and S1). Upon stimulation by LPS, iNOS was overexpressed, which was reversed with increasing concentrations of pre-treated GA-13-6. The reduction in iNOS expression was more pronounced when cells were pre-treated with GA-13-6 for 3 h. Interestingly, iNOS expression was slightly upregulated when cells were pre-treated with 25 μg/mL of GA-13-6, suggesting a potential temporal difference between the translation of actual proteins and the transcription of mRNA in the nucleus. On the other hand, the level of COX2 overexpression was not evident when cells were induced by LPS, while the reduction was more pronounced in cells pre-treated with GA-13-6 for 16 h. The overall expression levels of both iNOS and COX2 were higher when the macrophages were pre-treated with GA-13-6 for 16 h, aligning with the higher transcription levels of both mRNAs for samples pre-treated for a longer duration (See Fig 3a and 3b).

### Astilbin showed limited efficacy in the prevention of the immune responses in LPS-stimulated RAW 264.7 cells

**Fig 4:**
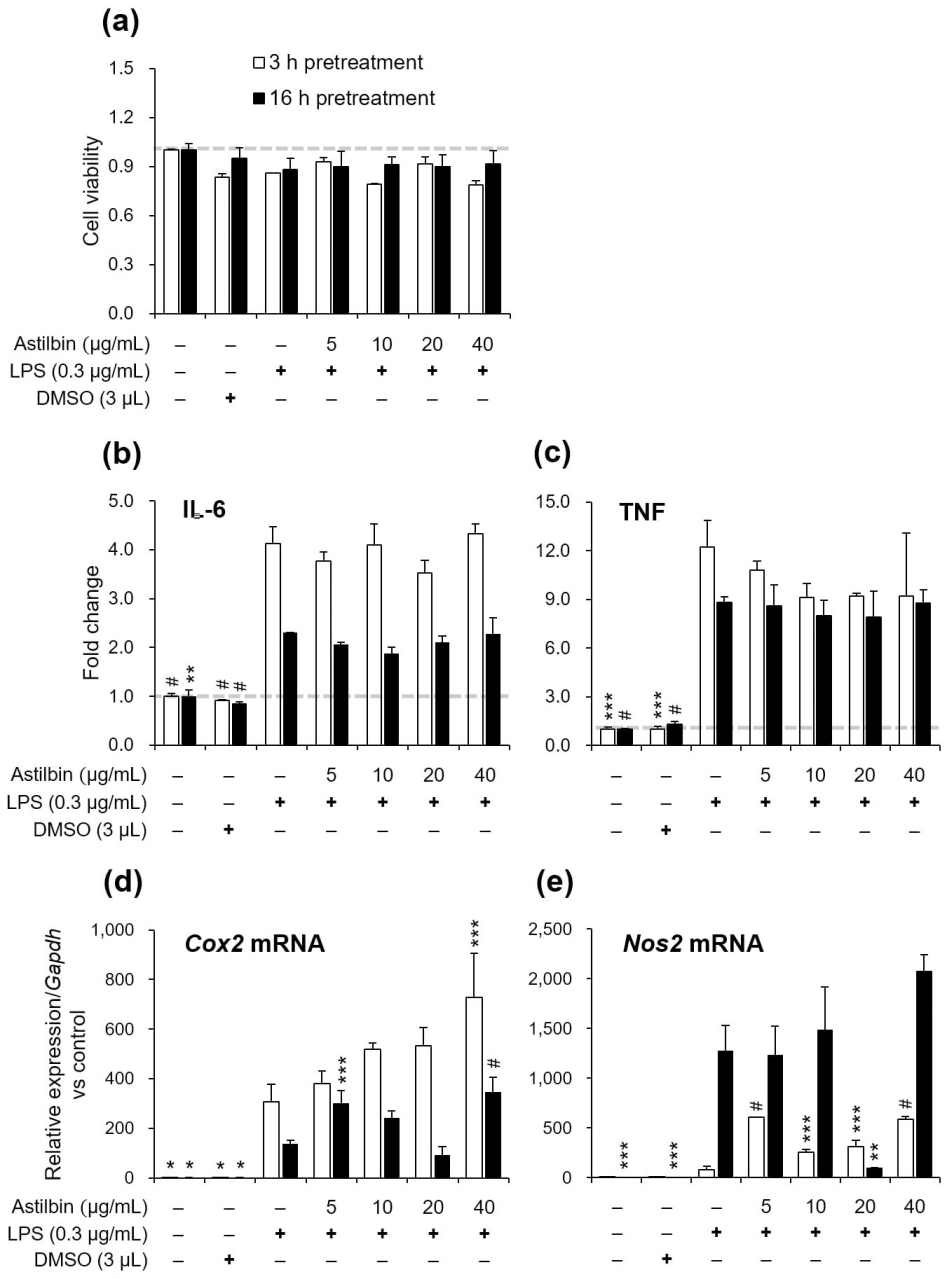
Effect of astilbin on cell viability of LPS-induced RAW 264.7 cells. The cells were pre-treated with astilbin (0, 5, 10, 20, 40 μg/mL) for 3 h (empty bars) and 16 h (filled bars), respectively, before LPS (0.3 μg/mL) treatment for 3 h. The cell proliferation was measured by MTT assay (a). The expression levels of IL-6 (b) and TNF (c) were determined by ELISA. The mRNA expression levels of *Cox2* (d) and *Nos2* (e) were normalized using the *Gapdh* gene. *p < 0.05, **p < 0.01, ***p < 0.005, ^#^*p* < 0.001 vs LPS-only group.

**Fig 5:**
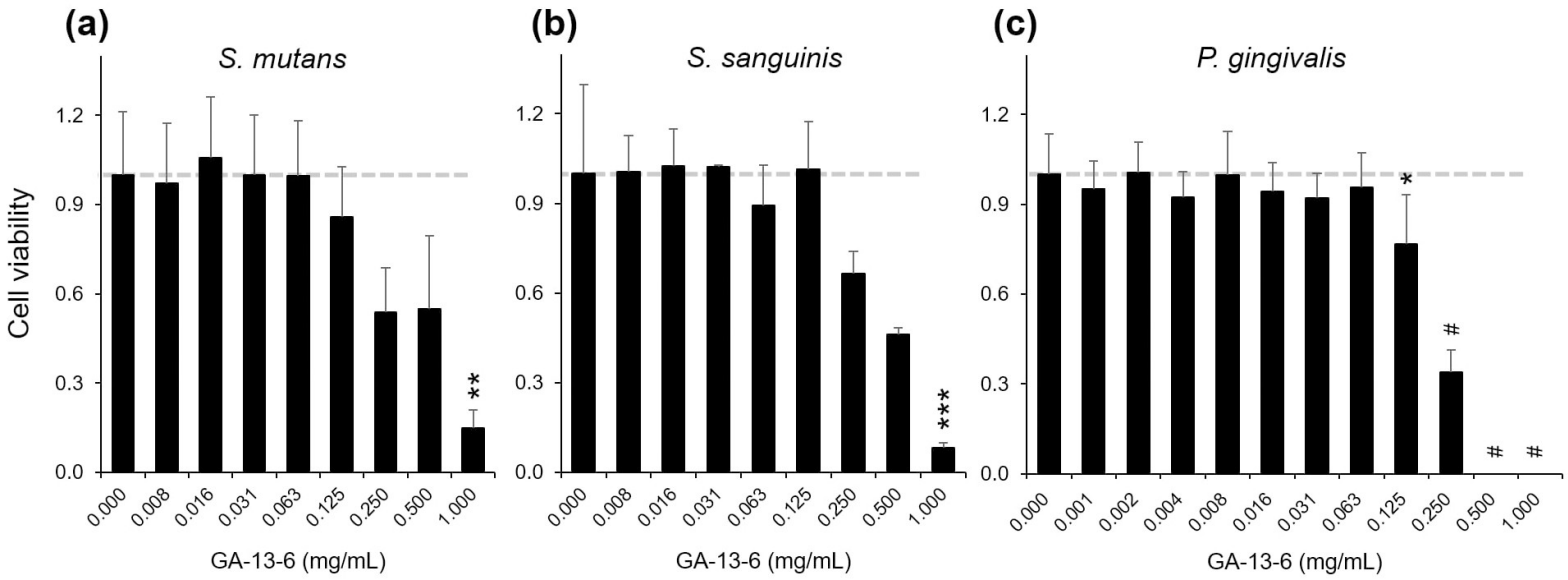
Antibacterial effect of GA-13-6 on oral key pathogens. GA-13-6 inhibited most strongly the growth of *P*. *gingivalis*, followed by *S*. *sanguinis* and *S*. *mutans*. Bacterial cell viability was determined by normalized OD_600_. *p < 0.05, **p < 0.01, ***p < 0.005, ^#^*p* < 0.001 vs control group (0.000 mg/mL of GA-13-6).

We proceeded to evaluate the anti-inflammatory effect of astilbin, a representative bioactive compound found in *A*. *chinensis* rhizome [23]. We anticipated that the efficacy of GA-13-6, as NCS that constitute heterogeneous compounds simultaneously, could differ from that of purified astilbin. Indeed, purified astilbin exhibited marginal cytotoxicity when applied to RAW 264.7 macrophages up to 40 μg/mL for 3 h and 16 h, respectively (Fig 4a). Unlike GA-13-6, however, the compound failed to reduce the activation of IL-6 and TNF (Fig 4b and 4c). On the contrary, the mRNA expression levels of *Cox2* and *Nos2* in LPS-induced cells significantly increased when cells were pre-treated with astilbin (Fig 4d and 4e). The induction of *Cox2* mRNA was dose-dependent when cells were pre-treated with astilbin for 3 h, which was disrupted with 16 h (Fig 4d). In contrast, the induction of *Nos2* mRNA was dose-dependent when cells were pre-incubated with astilbin for 16 h, except when 20 μg/mL of astilbin was used (Fig 5e). Both mRNA expressions were efficiently suppressed only when the macrophages were pre-incubated with 20 μg/mL of astilbin for 16 h, indicating a narrow window of optimal concentrations and pre-incubation time for this specific chemical to be effective.

### GA-13-6 inhibited the growth of major oral pathogenic bacteria

We next demonstrated the bacteriostatic antibacterial activity of GA-13-6 by determining minimum inhibitory concentrations (MICs) of three common oral pathogenic bacteria: *S*. *mutans*, *S*. *sanguinis*, and *P*. *gingivalis*. Our findings revealed that GA-13-6 efficiently inhibited the growth of pathogens in the following order: *P*. *gingivalis*, *S*. *sanguinis*, and *S*. *mutans* (Fig 5). The amount of GA-13-6 was insufficient to reach the MICs of *S*. *mutans* and *S*. *sanguinis*, resulting in estimated MICs of 2 mg/mL for both cells. The MIC of *P*. *gingivalis* was 0.5 mg/mL.

## Discussion

Generally, botanically-derived NCS represent a mixture of heterogeneous substances widely utilized as ingredients in various products under proper regulatory framework [31]. There are intrinsic variations in the chemical composition of NCS obtained from one unique genus and species owing to the differences in the region of growth, the annual variations in climate within the region, the part of the plant as source material, and processing methodologies, such as extraction, distillation, pressing, fractionation, purification, concentration, or fermentation [32].

While extracts of *A*. *chinensis* rhizomes have frequently been reported to possess anti-inflammatory [25,29], anti-tumor [33–37], and anti-platelet activities [38], studies on the extract of the inflorescence of *A*. *chinensis* have not yet been reported. In this study, we demonstrated that GA-13-6, as a patented NCS, exhibits anti-inflammatory properties as well as antibacterial effects on oral pathogenic bacteria such as *P*. *gingivalis*, *S*. *sanguinis*, and *S*. *mutans*. To this end, we collected three different parts (aerial, underground, inflorescence) of the plant from four different regions of South Korea in two different seasons. A total of 18 collected samples were initially screened for sample quality and quantity, followed by preliminary tests for cell cytotoxicity and anti-IL-6 activity. We selected GA-13-6 as a proprietary NCS candidate, which efficiently inhibited the activation of proinflammatory cytokines when RAW 264.7 macrophages were later induced by LPS treatment.

LPS-induced RAW 264.7 macrophages are commonly used for studies on inflammatory reactions, wherein NF-κB, a pivotal transcription factor in the nucleus, is activated upon LPS binding to TLR4 on the cell membrane [39,40]. NF-κB plays an important role in regulating inflammatory responses by upregulating the expression of proinflammatory cytokines and inflammatory mediators, such as TNF (formerly known as TNF-α), IL-6, iNOS, and COX2 [41,42]. TNF, a representative inflammatory cytokine, is secreted early in the immune response and is involved in the activation of inflammation and regulation of cell necrosis [43]. IL-6, also produced in the acute phase of inflammatory responses, contributes to host defense in both humoral and cellular immunity [44]. NO, an anti-inflammatory signaling molecule under normal physiological conditions, plays a significant role in the pathogenesis of inflammation when over-produced upon infectious and proinflammatory stimuli [45]. In such abnormal situations, iNOS increases to produce more NO by converting L-arginine to L-citrulline [46,47]. NO is also involved in the activation of COX2, leading to the simultaneous release of mediators, such as prostaglandin E2 (PGE_2_) and prostacyclin (PGI_2_), from the COX pathway [48,49]. Thus, selective inhibition of the iNOS pathway is an important strategy for controlling many chronic inflammatory diseases including, but not limited to, cognitive and cardiovascular diseases [50]. Particularly, when iNOS synthesis is induced by bacterial endotoxin LPS, the production of high levels of NO is delayed but prolonged [51], partially explaining our Western results wherein the level of iNOS protein expression is inconsistent with the level of mRNA expression (Fig 3b and 3c). The reason why LPS induction of the macrophages barely increases COX2 protein expression and why the expression levels of both COX2 and iNOS increase when the macrophages were pre-treated with GA-13-6 for a longer duration remains obscure, although the results suggest that GA-13-6 can counteract LPS-mediated immune responses in a dose-dependent manner (Fig 3c).

Previously, Gil *et al*. [28] demonstrated the anti-inflammatory effect of ACE from the rhizomes of the plant using LPS-stimulated RAW 264.7 macrophages and thioglycollate-elicited peritoneal macrophages from male C57BL/6 mice. They observed a decrease in the levels of inflammatory mediators (NO, iNOS, PGE_2_, and COX2) upon pre-incubation of the cells with ACE for 1 h before LPS (1.0 μg/mL) stimulation for over 24 h. In these experimental conditions, the degree of NO reduction was dose-dependent, where 25 μg/mL of ACE was sufficient to reduce the level of NO to half of the fully elevated level. The expression level of iNOS followed a similar pattern. However, the reduction levels of other proinflammatory cytokines (IL-6 and TNF) were limited and dose-independent. In contrast, we pre-incubated the cells with GA-13-6 for 3 h and 16 h to compare the effect of short- and long-term exposure before inducing inflammatory responses in RAW 264.7 cells using 300 ng/mL of LPS while maintaining cell viability. In our experimental conditions, the degrees of reduction of IL-6 and TNF were greater with short-term exposure to GA-13-6 (Fig 3b and 3c). By contrast, the secretion of NO was greater if the cells were pre-exposed to GA-13-6 for 16 h, resulting in a more drastic decrease as the concentration of GA-13-6 increased (Fig 3d). Likewise, the mRNA expression levels of both *Cox2* and *Nos2* diminished more substantially when the cells were exposed for 16 h in a dose-dependent manner. The protein expression levels, however, remained higher with 16 h pre-treatment of the macrophages with LPS (Fig 3), indicating that the half-life of the mRNA is shorter than that of the translated proteins, and the longer the incubation, the more the protein accumulation. Collectively, GA-13-6 exhibited no less anti-inflammatory effect than ACE of the underground parts in LPS-induced macrophages.

Astilbin, one of the major active flavonoids isolated from the rhizome of *A*. *chinensis* [52–54], is also found in numerous plants and processed foods, such as wines, champagnes, and turtle jelly [55]. Its anti-inflammatory activity has been demonstrated in various studies involving T helper 17 (Th17) cells in a psoriasis-like mouse model [56], HaCaT cells and a psoriasis-like guinea pig model [26], an adjuvant-arthritis rat model [52], high glucose-induced glomerular mesangial cells [23], T- and B-cells in lupus mice models [57], mouse J774A.1 macrophages [58], human chondrocytes [27], and osteoarthritis mouse [27] and rat models [59]. However, in our study, astilbin exhibited no inhibitory effect on the production of inflammatory mediators and proinflammatory cytokines in LPS-induced RAW 264.7 macrophages (Fig 4). Interestingly, astilbin isolated from *Smilax corbularia* was reported to have no inhibitory effect on NO production while blocking PGE_2_ release in RAW 264.7 cells induced by 1.0 μg/mL of LPS for 24 h [54]. Additionally, astilbin from the rhizome of *Smilax glabra* was reported to inhibit the production of NO and TNF but not IL-6 in RAW 264.7 cells induced by 1.0 μg/mL of LPS for 20 h [53]. Given that flavonoids, commonly found in photosynthesizing plants, generally possess anti-inflammatory activity [55], the inconsistency in results among research groups may stem from differences in cell lines, such as RAW 264.7 cells versus others, and the amount and duration of LPS treatment. It should be noted, however, that GA-13-6, as an NCS, comprises a variety of functional flavonoids [19] that together yield stronger anti-inflammatory activity than a single compound can [54].

Periodontal disease is associated with a variety of bacteria and the biofilms they form that can cause damage to the periodontal support structure, which is closely linked to many systemic diseases [2,60]. *P*. *gingivalis* serves as a keystone pathogenic bacterium in the onset of periodontal disease [61]. Recent studies have confirmed the close relationship between this bacterium and systemic diseases including cancer, cardiovascular disease [62–66], diabetes [67,68], rheumatoid arthritis [69], and Alzheimer’s disease [11–14,70–73]. *P*. *gingivalis* produces several potential virulence factors, such as gingipain proteases, outer membrane vesicles (OMVs), LPS, capsule, and fimbriae [13,14,72–75]. Among these, gingipain proteases (Rgp and Kgp) are essential for its survival while simultaneously acting as primary virulence factors [72]. Specifically, gingipains directly influence gene expression associated with dementia in the brain [73]. While capsule and fimbriae facilitate physical interactions with host cells, LPS, and OMVs trigger intracellular proinflammatory signaling pathways [14,66]. Conversely, bacterial commensals or opportunistic pathogens, such as *S*. *sanguinis*, *Streptococcus gordonii*, and *Candida albicans*, may create a favorable environment for *P*. *gingivalis* pathogenesis when there is disruption in the balance of the bacterial community [74]. Considering that plant flavonoids can reduce inflammatory responses and inhibit bacterial growth [76], we hypothesized that GA-13-6 may contain various flavonoids and could suppress the growth of oral pathogens. As anticipated, GA-13-6 efficiently inhibits the growth of *P*. *gingivalis* as well as *S*. *sanguinis* and *S*. *mutans*, suggesting that GA-13-6 may prevent infection and suppress ensuing inflammation if any infections occur. The difference in the inhibitory efficacy of GA-13-6 between Gram-positive and Gram-negative bacteria as shown in Fig 5 could be attributed to differences in membrane structures, which can be disrupted by some flavonoids [77]. Thus, instead of using conventional antibiotics for treatment, which can lead to dysbiosis and antibiotic resistance, the use of NCS such as GA-13-6 may facilitate the restoration of healthy oral commensalism with minimal side effects.

In this study, we made an effort to collect three different parts of *A*. *chinensis* from a wide variety of regions in two different seasons and screened the optimal part of the plant that could effectively prevent the onset of cellular inflammation as well as the growth of oral pathogenic bacteria. Following the screening process, we first discovered that the ACE of the inflorescence of *A*. *chinensis*, GA-13-6, obtained during the flowering season, exhibited the best performance. We aimed to utilize GA-13-6 as NCS for the prevention and treatment of periodontal disease, a near-pandemic disease in the oral cavity, as well as widespread dental caries. As expected, GA-13-6 successfully suppressed both cellular inflammation responses and oral bacterial growth.

GA-13-6, an ethanol extract from *A*. *chinensis* inflorescence, efficiently suppressed the activation of proinflammatory cytokines and inflammatory mediators, such as TNF, IL-6, and NO, as well as the expression of COX2 and iNOS enzymes in LPS-stimulated RAW 264.7 macrophages. The anti-inflammatory efficacy of GA-13-6 surpasses that of purified astilbin, one of the major effective ingredients found in *A*. *chinensis*. The antibacterial effects of the extracts were also confirmed for the first time against prevalent oral pathogens, such as *S*. *mutans*, *S*. *sanguinis*, and *P*. *gingivalis*, indicating that GA-13-6 can inhibit bacterial infection in the oral cavity and suppress ensuing inflammatory responses. Further study is needed to identify the active ingredients of GA-13-6 responsible for the anti-bacterial efficacy and to define the selectivity of GA-13-6 for many other benign and harmful bacteria in the oral cavity.

## Supporting information

S1 Fig Western raw images and quantification analysis on the effect of GA-13-6 on protein expression levels of COX2 and iNOS in LPS-induced RAW 264.7 cells. (a) Triplicate western blot images. The third images were used for Fig 3c. M: Protein markers (inversely stained). *Non-specific bands. (b) Quantification of the western bands corresponding to COX2 and iNOS. *p < 0.05 vs LPS-only group.
(TIF)

S1 Dataset Complete manuscript raw dataset providing cell viability tests, immunoassays, qPCR and western assays, bacterial growth inhibition assays, and ANOVA analyses. (XLSX)
