# Supplementary figures and images for: Ethanol extract from Astilbe chinensis inflorescence suppresses inflammation in macrophages and growth of oral pathogenic bacteria
Source: PLoS One. 2024 Jul 3;19(7):e0306543. doi: 10.1371/journal.pone.0306543 (PMC11221678; doi:10.1371/journal.pone.0306543)

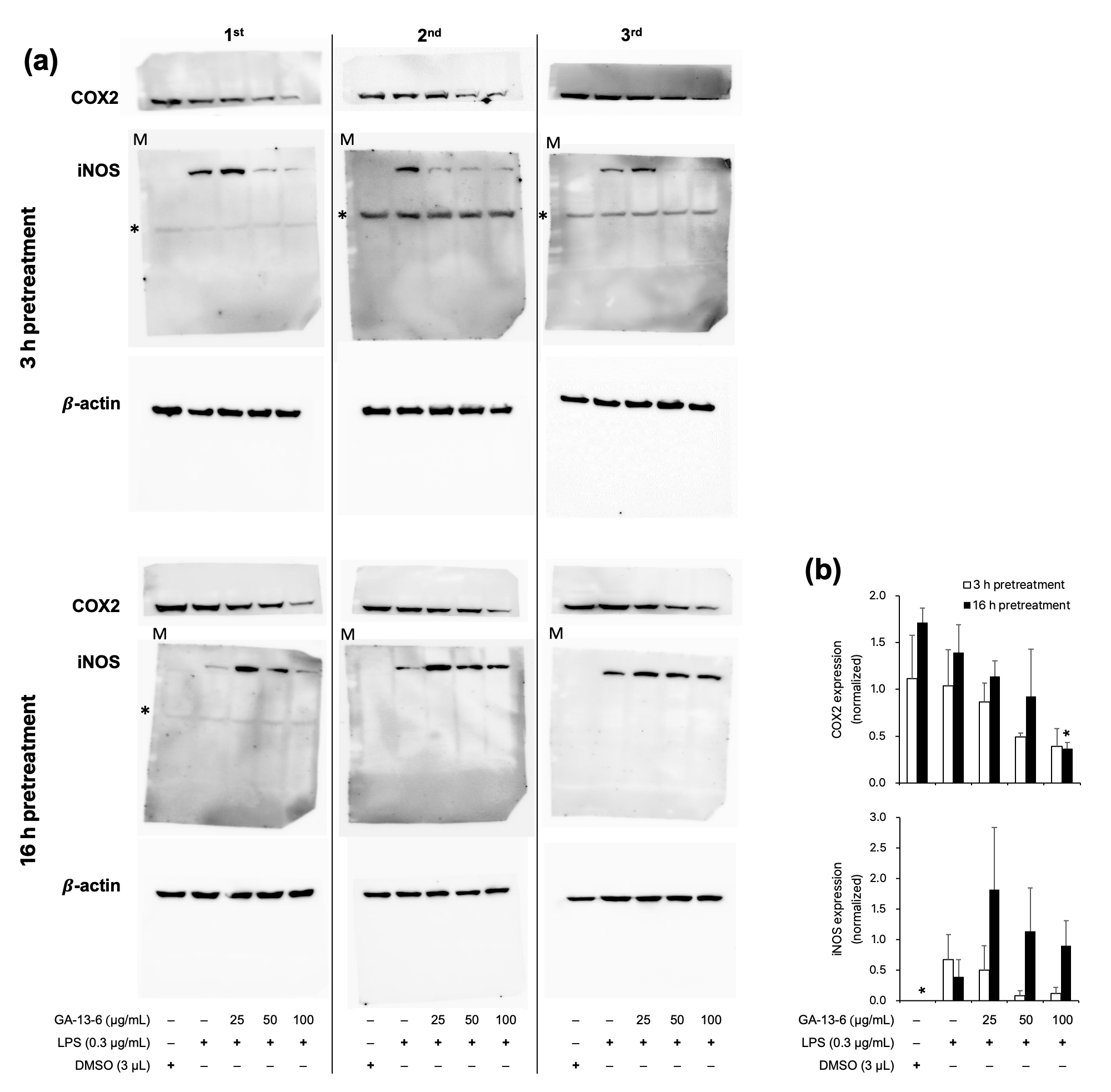

Supplement: S1 Fig — (a) Triplicate western blot images. The third images were used for Fig 3c. M: Protein markers (inversely stained). *Non-specific bands. (b) Quantification of the western bands corresponding to COX2 and iNOS. *p < 0.05 vs LPS-only group. (TIF) [file pone.0306543.s001.tif]
